# Supplementary material for: Molecular Phylogeny and Morphology Reveal Cryptic Species in the Cordyceps militaris Complex from Vietnam
Source: J Fungi (Basel). 2023 Jun 15;9(6):676. doi: 10.3390/jof9060676 (PMC10302822; doi:10.3390/jof9060676)
Supplement: Supplementary file 1 [file jof-09-00676-s001.zip › jof-2399972-supplementary.pdf]

**Table S1. Primers used in the study.**

| Gene | Primer name | Primer sequence (5'-3') | Reference |
|---|---|---|---|
| nr*SSU* | nrSSU-CoF | TCTCAAAGATTAAGCCATGC | [16] |
| | nrSSU-CoR | TCACCAACGGAGACCTTG | |
| nr*LSU* | LR5 | ATCCTGAGGGAAACTTC | [17,18] |
| | LR0R | GTACCCGCTGAACTTAAGC | |
| *TEF* | EF1α-EF | GCTCCYGGHCAYCGTGAYTTYAT | [12,19] |
| | EF1α-ER | ATGACACCRACRGCRACRGTYTG | |
| *RPB1* | RPB1-5'F | CAYCCWGGYTTYATCAAGAA | [12,19] |
| | RPB1-5'R | CCNGCDATNTCRTTRTCCATRTA | |
| *RPB2* | RPB2-5'F | CCCATRGCTTGTYYRCCCAT | [12,19] |
| | RPB2-5'R | GAYGAYMGWGATCAYTTYGG | |
